# Supplementary material for: Role and mechanism of NCAPD3 in promoting malignant behaviors in gastric cancer
Source: Front Pharmacol. 2024 Apr 22;15:1341039. doi: 10.3389/fphar.2024.1341039 (PMC11070777; doi:10.3389/fphar.2024.1341039)
Supplement: Supplementary file 11 [file DataSheet2.ZIP › GSEA/immunologic/my_analysis.Gsea.1599462003817/GSE14000_UNSTIM_VS_4H_LPS_DC_UP.html]

Details for gene set GSE14000\_UNSTIM\_VS\_4H\_LPS\_DC\_UP[GSEA]

|  || Dataset | filtered\_dataset.sample\_info.cls#WT\_versus\_NCAPD3\_MUT |
| Phenotype | sample\_info.cls#WT\_versus\_NCAPD3\_MUT |
| Upregulated in class | WT |
| GeneSet | GSE14000\_UNSTIM\_VS\_4H\_LPS\_DC\_UP |
| Enrichment Score (ES) | 0.4246575 |
| Normalized Enrichment Score (NES) | 1.92468 |
| Nominal p-value | 0.01048951 |
| FDR q-value | 0.60137796 |
| FWER p-Value | 1.0 |
Table: GSEA Results Summary

  

Fig 1: Enrichment plot: GSE14000\_UNSTIM\_VS\_4H\_LPS\_DC\_UP      
 Profile of the Running ES Score & Positions of GeneSet Members on the Rank Ordered List

  

| SYMBOL | TITLE | RANK IN GENE LIST | RANK METRIC SCORE | RUNNING ES | CORE ENRICHMENT || 1 | 388969 | C2orf68 | 10 | 1.097 | 0.0816 | Yes |
| 2 | 3954 | LETM1 | 47 | 0.924 | 0.1305 | Yes |
| 3 | 286101 | ZNF252P | 136 | 0.758 | 0.1285 | Yes |
| 4 | 613 | BCR | 180 | 0.695 | 0.1538 | Yes |
| 5 | 54801 | HAUS6 | 219 | 0.660 | 0.1798 | Yes |
| 6 | 6573 | SLC19A1 | 231 | 0.650 | 0.2245 | Yes |
| 7 | 6850 | SYK | 256 | 0.625 | 0.2578 | Yes |
| 8 | 25974 | MMACHC | 265 | 0.621 | 0.3024 | Yes |
| 9 | 56910 | STARD7 | 335 | 0.573 | 0.2991 | Yes |
| 10 | 132001 | TAMM41 | 524 | 0.460 | 0.2007 | Yes |
| 11 | 147015 | DHRS13 | 559 | 0.441 | 0.2120 | Yes |
| 12 | 79707 | NOL9 | 560 | 0.441 | 0.2477 | Yes |
| 13 | 27349 | MCAT | 574 | 0.436 | 0.2736 | Yes |
| 14 | 26100 | WIPI2 | 601 | 0.423 | 0.2891 | Yes |
| 15 | 23404 | EXOSC2 | 609 | 0.418 | 0.3179 | Yes |
| 16 | 23212 | RRS1 | 681 | 0.387 | 0.2981 | Yes |
| 17 | 4507 | MTAP | 690 | 0.384 | 0.3234 | Yes |
| 18 | 22877 | MLXIP | 701 | 0.377 | 0.3467 | Yes |
| 19 | 55759 | WDR12 | 731 | 0.359 | 0.3549 | Yes |
| 20 | 27300 | ZNF544 | 759 | 0.343 | 0.3632 | Yes |
| 21 | 51118 | UTP11L | 773 | 0.336 | 0.3810 | Yes |
| 22 | 90231 | KIAA2013 | 787 | 0.330 | 0.3984 | Yes |
| 23 | 9343 | EFTUD2 | 818 | 0.305 | 0.4015 | Yes |
| 24 | 26608 | TBL2 | 821 | 0.304 | 0.4247 | Yes |
Table: GSEA details [plain text format]

  

Fig 2: GSE14000\_UNSTIM\_VS\_4H\_LPS\_DC\_UP      
 Blue-Pink O' Gram in the Space of the Analyzed GeneSet

  

Fig 3: GSE14000\_UNSTIM\_VS\_4H\_LPS\_DC\_UP: Random ES distribution      
 Gene set null distribution of ES for **GSE14000\_UNSTIM\_VS\_4H\_LPS\_DC\_UP**
